# Is the use of sentient animals in basic research justifiable?

**DOI:** 10.1186/1747-5341-5-14

**Published:** 2010-09-08

**Authors:** Ray Greek, Jean Greek

**Affiliations:** 1 Americans For Medical Advancement, 2251 Refugio Rd, Goleta, CA 93117, USA

## Abstract

Animals can be used in many ways in science and scientific research. Given that society values sentient animals and that basic research is not goal oriented, the question is raised: "Is the use of sentient animals in basic research justifiable?" We explore this in the context of funding issues, outcomes from basic research, and the position of society as a whole on using sentient animals in research that is not goal oriented. We conclude that the use of sentient animals in basic research cannot be justified in light of society's priorities.

## Introduction

The purpose of this paper is to explore the use of sentient animals in basic research. (We realize humans are animals but will use the word *animal *to mean nonhuman animal in this review.) We ask the question, "Is the use of sentient animals in basic research justifiable?" The reason we ask the question this way is that there is evidence that society has decided that if sentient animals can be used to predict human response to drugs and disease, then using them is acceptable. However, such use is not scientifically tenable, as animals cannot predict human response [1-27]. (See references 1 and 2 for reviews that include the theory behind this position and the empirical evidence supporting it. See references 3-27 for analysis of selected examples. We fully understand the contentious nature of our statement that animals cannot predict human response to drugs and disease but our defense of that statement is in references 1 and 2, not in this paper.) As a result, the questions that arise are: "What of using sentient animals in research that is recognized as curiosity-driven rather than goal-oriented? What factors should be considered when using sentient animals in such an endeavour? What would an informed society think justifies the use of sentient animals in research in general?"

In this essay, we show that: 1) basic research by definition is *not *designed to lead to cures; 2) in a vast majority of cases it does not; and 3) we show that society is not comfortable with this situation. We view this paper as a syllogism. IF society is not comfortable, or does not condone, using sentient animals in research that does not lead to cures and IF basic research is just that kind of research THEN society does not condone using sentient animals in basic research.

If basic research is defined as research that is *not *designed to predict human response to drugs or disease and is curiosity-driven, then what is purpose of taking the readers' time to explore the use of sentient animals in basic research? Is not the outcome already known? In reality, the previous points are very contentious, as is the conclusion, and thus the point of this essay is to take the reader through the major considerations. The authors started this discussion along with Niall Shanks in the article, "Are animal models predictive for humans?" [1] This essay is part two of our examination of the issue of using animals in research and in science in general.

This entire topic is very emotional and contentious and has many facets. There are many additional questions that can and eventually should be addressed. For example:

1. What kind of basic research can be performed without using animals and what are relative benefits and costs?

2. What was the role of animals in past scientific and medical breakthroughs? (This is not an easy question to answer. The exact history of how breakthroughs and discoveries happened is more similar to assembling a jigsaw puzzle than a straightforward example of A led to B led to C.)

3. Could breakthroughs that used animals have happened without using animals? If such breakthroughs could not have occurred without animals, during that particular era in history, why was this the case? Did advances in science and or engineering subsequently occur that would have allowed the discovery or breakthrough to have been made later without the use of animals? What would the consequences have been of a later date for the breakthrough?

4. How are animals used in the totality of research and science and what are the scientific merits of these uses? For example, in the review article "Are animal models predictive for humans?" [1] and the book *Animal Models in Light of Evolution *[2] the authors outline nine ways animals are used in science and advocate for the position that seven out of the nine ways are scientifically viable. By dividing the use of animals into categories, as we are doing in this essay, the topic is not only made more manageable but also allows for more precision in the arguments. Also, sweeping generalizations are avoided. Scientifically viable use of animals in one of the nine categories cannot be used to justify the ways animals are used in other categories.

5. Should there be a concerted effort by scientists to explain the value of basic research *for knowledge sake alone*? Would the position of society on using sentient animals in basic research change if society believed, like many scientists, that research with sentient animals is justified solely on the basis of achieving more knowledge?

6. What barriers exist to replacing animals in research? What role do Institutional Review Boards and Institutional Animal Care and Use Committee play in this? Do they help or hinder? How available is human tissue as opposed to animal tissue and why?

7. What does *research about research *reveal? It is the opinion of the authors that society needs much more research about research. What works and what does not? What has the highest rate of return? Is the division between the National Institutes of Health (NIH) and the National Science Foundation (NSF) working out as planned or should the process be changed? What types of research are under funded and what types are over funded?

8. Last in this list (but this is by no means an exhaustive list) is the issue of ethics. Can humans use animals in whatever way we see fit? Why do European counties requires more justification of the ethical cost:benefit ratio than the US? Are some sentient animals more worthy of consideration than others?

All of the above are part and parcel of the general topic of using animals in research and science. As we did in the essay "Are animal models predictive for humans?" [1], in this essay we break the problem down into one manageable question while acknowledging that the issue *per se *is much larger and that this essay is but one component of many.

## Societal Norms

Philosophies of life vary considerably, especially where using animals in research is concerned. Some hold that animals should be used regardless of sentience or societal concerns. Derbyshire is representative of many in the basic research community when he states:

Ultimately, we cannot have it both ways. It is not possible to advocate animal welfare and at the same time give animals untested drugs or diseases, or slice them open to test a new surgical procedure. The three Rs [the notion that the number of animals used should be **R**educed, the procedures **R**efined to decrease pain, and animals as a whole should ultimately **R**eplaced with nonanimals] encourage a focus on animal welfare that is both unrealistic and dishonest. Regardless of any beliefs about the value of animals, if you engage in activities that are invasive or lethal to animals or if you control their reproduction, their living space and their habits, you are expressing a de facto belief that animals are sufficiently different from humans to make such activities justifiable. Scientists are keen to defend themselves against accusations of cruelty by promoting their allegiance to the three Rs but forget that the real reason for animal experimentation is to advance the welfare and understanding of humanity. Advancing human understanding requires the freedom to do more animal research, and often with higher species, and is incompatible with continued support for the three Rs [28].

Others hold a different view. A poll conducted by the Pew Research Center and the American Association for the Advancement of Science (AAAS) and released on July 9, 2009 revealed that only 52% of the nonscientist general public supported the use of animals in scientific research [29]. (It should be noted here that most polls on this subject, like the Pew/AAAS poll have been of the *either or *variety: "Do you support or reject the use of animals in research?" Such polls have thus not allowed the respondent any flexibility in the response or any nuance in his position. From our perspective these polls are suboptimal hence should be used as an acknowledged inexact metric or for tracking purposes. We will not be relying on them here. Suffice it to say, more polls that allow for a division of the use of animals in science and research are needed.) Better polls have asked more specific questions and have consistently revealed that society in general condones using animals in research when they think it will lead to life-saving treatments but not when they think it curiosity-driven. For example, in 1999, MORI conducted a poll in association with *New Scientist *that was published in *New Scientist *on May 22, 1999 [30]. When respondents were asked whether they favored using animals, 24% answered yes while 64% said no. But the pollsters then broke the questions down into several categories. For example, when respondents were questioned about experiments in which mice would be subject to pain, illness or surgery, 61% disapproved using them in order to study how the sense of hearing works, but only 32% disapproved of using the mice to ensure a new drug to cure childhood leukemia is safe and effective. When monkeys were substituted for mice the disapproval went from 64% to 75% and 32% to 44%, respectively.

The above suggests that more detailed questions reveal more about society's attitudes than simple *either or *questions. It appears to us that the more informed society is--*vis-à-vis *more precise questions--the more uncomfortable they are over using sentient animals for non-goal oriented research. Anecdotally, we see the most discomfort using animals like nonhuman primates, dogs, and cats--animals that society is ether exposed to on a daily basis or relates to as being *like us*. This raises many questions, one of which is: "How important is current biomedical basic research is in leading to treatments?"

Societies built on the principles of so-called *Western philosophy *(the United States, Europe and so forth) appear to be uncomfortable with sentient animals being used in basic research; basic being defined as research not designed to lead to cures. This theme continues with Giles writing in *Nature*:

In the contentious world of animal research, one question surfaces time and again: how useful are animal experiments as a way to prepare for trials of medical treatments in humans? *The issue is crucial, as public opinion is behind animal research only if it helps develop better drugs*. Consequently, scientists defending animal experiments insist they are essential for safe clinical trials, whereas animal-rights activists vehemently maintain that they are useless [31]. (Emphasis added.)

The Institute for Laboratory Animal Research [[32]a] and other proponents of using animals in research [33] have views similar to Giles. An editorial in *Nature *in 2009 reinforced the above stating: "Animal-research policies need to be guided by a moral compass--a consensus of what people find acceptable and unacceptable." [34] It should be noted here that this position is somewhat at odds with what the late animal campaigner Henry Spira claimed. Spira thought that as long as society accepted *eating *animals as morally proper, it would have no problem with experimenting on them. This is important to our discussion as, in making his claim, Spira ignored the fact that many of the founders of the various antivivisection societies ate meat and that members of antivivisection societies today do as well. The *Nature *comment is closer to the mark--people can be and are inconsistent and some things bother them more than others. Such is the reality of life.

Many if not most in our *Western *society allow that sentient animals deserve some moral consideration when discussing their use in research and journals like *Nature *acknowledge this. (We realize that society is not monolithic and that opinions vary on almost all issues including this one. Nevertheless, judging from polls and comments in scientific journals it appears that the US and Europe, at least, are composed of individuals that, on the whole, are not comfortable with sentient animals being used in curiosity driven research.) There is a cost:benefit analysis to be done here--the cost being the suffering of sentient animal--and some in society have performed this analysis and are not comfortable with using animals in basic research but are comfortable with using them in other ways. (There is another *cost *and that is the relative merit of basic research on the whole, using animals or not, as opposed to spending our limited research budget on clinical research or other areas of research. However, as this is not our topic, we will leave it for another.) It is this view--the cost:benefit analysis that values cures but not curiosity-driven research--that we will assume when discussing animal use for basic science.

## Definitions

Since we are concerned with two concepts, sentience and basic research, we will take a moment to better define or describe them. We have already referred to basic research as being research that is not designed to lead to cures but we need to lend some support to this definition. Basic research has also been called basic science research, curiosity-driven, blue-sky research, pure research, and fundamental research [32,35-38]. We will refer to it as basic research. Basic research can be variously defined and what researchers mean when they say *basic research *varies considerably, but the following definition is representative. The Organisation for Economic Cooperation and Development said basic research is:

Experimental or theoretical work undertaken primarily to acquire new knowledge of phenomena and observable facts without any particular application or use in view. It is usually undertaken by scientists who may set their own agenda and to a large extent organise their own work [39].

Francis Bacon [40], Claude Bernard[41], and JJ Thompson[[42], the discoverer of the electron, The National Environment Research Council [38], Braben [43], and others [[32]b], [35] agree with the thrust of the above. Arthur Kornberg stated in an Editorial in *Science *in 1995:

We are urged: Do strategic basic research! Do targeted basic research! How can we make clear the oxymoronic nature of these terms? [44]

While the above does not ensure that the definition we are using is universally acceptable, it does make clear the distinction between applied, goal-oriented research that, in our view, is synonymous with *predictive research *and research that is *not*, by its nature, predictive for humans.

Let us be very clear on the importance of basic research in science, historically. Because of basic research, many of the most important breakthroughs in physics, chemistry, and biology happened. Basic research has been very important to scientific advancement.

Discoveries and inventions derived from basic science research include:

• The discovery of DNA

• Basic biochemistry, such as the Krebs cycle

• The periodic table of the elements

• The mass spectrometer

• Transistors

• Computer circuits

• X-rays

• Electrons, protons, and neutrons

• Nuclear power

• Electromagnetic waves

• Induction coils in automobiles

• Global Positioning Satellite system

Basic research does not, however, necessarily involve using sentient animals. Basic research can be conducted using nonsentient animals, on a computer, in a physics or chemistry lab, doing thought experiments, or in myriad other ways. Virtually all basic research in chemistry and physics (that led to the discoveries listed above) does not involve using sentient animals and many of the greatest discoveries that reduced the burdens of illness and disability came from these two fields. For example, CT scans, PET scans, radiographs, cathode rays, thermionic valves, x-ray crystallography, nuclear magnetic resonance and MRI scanners, radioactive implants, the ultracentrifuge, methods for preparing pure enzymes and viruses, the chemistry of hormones, protein electrophoresis, chromatography, electron microscopy, mass spectroscopy, and many more were all the results of basic research in physics and chemistry. These discoveries and the technology represented by these discoveries have probably gone further to alleviate suffering than most other breakthroughs.

We are by no means questioning the value of basic research *per se *in science in general. (We do discuss the fact that the relative importance of basic research in the biomedical sciences *is *being questioned with regard to how much money should be directed to it as opposed to more clinically oriented research.) By definition, anything that leads to more knowledge is valuable if one values more knowledge. Many argue that *any *knowledge gained is worthwhile and no one can deny that even today knowledge is being gained from using sentient animals. There are certainly experiments currently conducted on, for example, nonhuman primates for the purposes of studying neurophysiology that cannot be performed upon humans. We acknowledge that scientific knowledge can be and is being advanced by studying sentient animals in laboratories. This fact is not in dispute in the paper. Rather, we are discussing the necessity or the cost:benefit ratio, *as appreciated by society*, of using sentient animals in such research in the biomedical sciences.

Basic biological research has traditionally studied life at the most basic level; what the cell is, what it is made of, what distinguishes life from nonlife, what everything is built of and so forth. In applied research, the scientists usually want to make something commercially viable. There is no doubt that research is a continuum ranging from basic to applied and it is not always easy to categorize a specific research project. But, based on the definitions above, one thing remains certain: *Basic research makes no claims of applicability*.

Historically, animal use in research was synonymous with basic research. It was easy to dissect or vivisect animals without any particular end in mind. If you were curious about a phenomenon or wanted to learn more about life in general, animals could be used. For example, Claude Bernard's research with animals was largely basic science research. This approach was largely successful when scientists wanted to learn the very fundamentals of life. After all, monkeys, frogs, mice, and humans have much in common. But research today and the practice of medicine today focuses on the differences between individual humans [45-61] not the commonalities between humans and other animals. This has implications for our theme.

The second term that needs to be defined is sentience. *Sentient*, like *basic research*, can be variously defined, including:

• having sense perception;

• consciousness;

• experiencing sensation or feeling;

• responsive to or conscious of sense impressions;

• aware;

• finely sensitive in perception or feeling;

• able to experience physical and possibly emotional feelings;

• having the capacity to receive sensations;

• able to perceive.

For the purposes of this paper, exactly which animals are sentient and which are not is immaterial. Most people will agree that dogs, chimpanzees, and mice are sentient while most will also agree that fruit flies, worms, and members of Cnidarian are not. The controversy we are addressing is whether society approves of sentient organisms *per se *being used in basic research, not exactly which animals occupy this category. Exactly which animals are sentient can be discussed after the concept we are exploring has been settled and indeed is already being discussed in many books and journals.

The issue of whether sentience confers moral consideration has also been addressed elsewhere and we refer the reader to those arguments [62-66]. Very briefly, such arguments state that sentience is the only morally relevant trait that all current recipients of moral consideration have in common; hence any sentient individual should be a moral recipient. This is called the *argument for moral consistency*. While society *per se *cannot articulate the argument for moral consistency, they certainly have a sense of the concept.

## Julius Comroe and Robert Dripps

Using animals in basic research is a division of basic research in general. Examining the value of basic research in biomedical research is a good place to begin our discussion.

Susan Hockfield, president of the Massachusetts Institute of Technology, wrote in an editorial for *Science*:

U.S. federal investments in basic research transformed life and commerce in the 20th century. They sent us to the Moon and beyond, revolutionized, helped to feed the planet, reinvented work processes, and drove the remarkable economic growth of the post-1950 s era in the United States. These advances and more grew out of the convergence between engineering and the early 20th-century discoveries in the physical sciences. The United States can anticipate comparable world-changing innovations in the 21st century if we adapt our education and research funding strategies to capitalize on new opportunities emerging at the convergence of the life sciences with the physical sciences and engineering [67].

Basic research in the United States began in earnest after World War II. This was due at least in part to the engineer Vannevar Bush, director of the Office of Scientific Research and Development. Bush wrote a report for president Roosevelt stating that "new knowledge can be obtained only through basic scientific research." [68]

This marked a turning point in research funding. In the 19th century, most research had been privately funded, with industrial and government funding increasing in the 20th century. After this report, government funded research, as opposed to privately funded research, became the norm. In the 19th century, research was expected to produce results. Not all did, and some was funded without such expectations, but overall the funders expected practical results. As a result of this report, the US government, in 1950, formed the National Science Foundation which has funded basic research ever since. (Today, NIH as well as other government agencies and charities also fund a large amount of basic research.) This emphasis on basic research spread across the Atlantic and has been the standard worldwide ever since.

Has the value of basic research in *current-day medicine *been proven? Everyone has anecdotes, sometimes many, to support their view that basic research, especially basic research using sentient animals, is vital for medical science to advance. But are there scientific data to support this view? The current emphasis on basic research in medicine, as opposed to applied research, grew out of a U.S. Defense Department study published in 1967 in *Science *that concluded that research performed with an end in mind was far more effective in improving a technology than research performed with no goal in mind, e.g., basic research [69]. This study led then-president Johnson to state: "[A] great deal of basic research [in medicine] has been done ... but I think the time has come to zero in on the targets -- by trying to get our knowledge fully applied ... We must make sure that no life-saving discovery is locked up in the laboratory [70]."

This perceived negative attitude about basic research led respiratory physiologist Julius Comroe and anesthesiologist Robert Dripps to conduct a survey concerning medical discoveries. The classic justification for basic research comes from that study published in 1976 [71]. Their paper "Scientific Basis for the Support of Biomedical Science," purported that 41 percent of all articles judged to be essential for later clinical advances in cardiovascular and pulmonary medicine and surgery, were not clinically oriented at the time they were conducted and that 62 percent of key articles were the fruits of basic research. This appears to be strong evidence that basic and translational research with animals is key to finding cures and was in fact seized upon by other countries. As noted by Grant et al.:

Since that analysis, support for basic research has increased in the G7 countries. In the UK, Research Council expenditure on basic research has increased from a low of £444 million (or 42 per cent of total civil R&D) in 1991/1992 to £769 million (or 61 per cent of total civil R&D). Although it would be difficult to argue that Comroe and Dripps were directly responsible for a strategic shift (or drift) in the type of science supported by research funders, their arguments are often cited (albeit at times implicitly) in support of increased funding for basic biomedical research [72].

A PubMed search (conducted on August 17, 2010) revealed 22 citations for the 1976 Comroe-Dripps paper. We believe this is very significant. Our claim that basic research, specifically basic research using sentient animals, is the accepted standard for advancing knowledge that will eventually be used to develop treatments, is supported by the paucity of references. The value of Comroe-Dripps is simply not questioned despite the far-reaching ramification as noted by Grant et al. above. Only recently [72] has the conclusion of Comroe-Dripps begun to be seriously questioned.

At this point in time we begin to see a dichotomy in how scientists explain the value of basic science research to society. Because of comments by Johnson and others and the great advances in applied research, some in the basic research community began feeling pressure from society to justify their research on grounds other than knowledge for knowledge sake. This break from the past has direct implications for our discussion. Society was already hinting that there are limits to what it would fund in terms of knowledge for knowledge sake. It is our contention that much current basic research is done under the guise of applied research because it increases the likelihood that the project will be funded by a granting institution [2]. For example, Freeman and St. Johnston in 2008:

Many scientists who work on model organisms, including both of us, have been known to contrive a connection to human disease to boost a grant or paper. It's fair: after all, the parallels are genuine, but the connection is often rather indirect [73].

Using animals as causal analogical models [74] or predictive models is not basic research; it is applied research. The real crux of the argument for some seems to be: "Give us money for basic science research using sentient animals because our research is predictive for humans" [[2]b]. When such research turns out *not *to be predictive, however, they state: "Our research is basic research so it is not supposed to be predictive." Even those who admit that basic research using sentient animals is not predictive hide under the umbrella of "animal models really are predictive" to increase their likelihood of obtaining grant money [[2]b].

(We should here point out that we do not believe the scientist-reader so naïve as to *not *understand what we are referring to. We, and we feel sure the scientist-reader, are very aware that in order to gain funding from institutions like NIH, the applicant is under pressure to show that the research in question ties in directly with a human disease [unpublished observations]. The applicant is under pressure to turn, what has been considered basic research, into applied research. The problems with this condition are numerous, nevertheless are not part of our considerations at present. Suffice it to say we are attempting to use words and phrases, like *basic research*, that have meaning consistent with reality as opposed to the meanings attributed to them in the grant-funding process.)

Comroe and Dripps were basic research and animal testing enthusiasts. They had criticized President Johnson's administration for coming out in favor of applied, not basic, research. They also criticized the first heart transplant surgeons for failing to publicly state that the operation, in their opinion, was only possible secondary to the use of animals in basic research [71,75-77]. Comroe had also written a critique of medical progress stating that all major discoveries had been a result of basic research involving animals [76,77]. Comroe was also critical of clinical research:

Let's not live in constant fear of the great god Randomization [clinical research], his (or her) appetite is huge, and, if fed continuously, could consume much of the nation's research dollars and personnel, and even the lives of patients [78].

The above statement is still quoted as a reminder that many have historically held and still hold clinical research in disdain. Silverman in 2004:

At the time of the 1969 debate [regarding optimal oxygen levels in premature babies], I found it hard to understand why those who spoke against a formal controlled trial won the "methods" debate so easily (the power of RCT [randomized controlled trial] format had been firmly and widely established following the famous clinical trials in Britain in the 1940 s and 1950s). But I had underestimated the influence of the counteroffensive mounted in the U.S. by prominent laboratory-oriented researchers. For example, one celebrated leader wrote ...

Here, Silverman quotes Comroe (the "great god Randomization" quote referenced above) then continues:

These dismissive comments concerning the use of statistical methods in clinical studies was a reminder of the disturbing split in outlook about how the medical profession should go about solving puzzles that turn up on the wards [79].

Comroe and Dripps surveyed the "scientific community" to determine which discoveries were important. They sent a number of surveys (some have estimated approximately one-half) to scientists performing basic science experiments. Not surprisingly, these scientists concluded that basic science animal studies had been invaluable. As the then-assistant editor and future editor of the *British Medical Journal *pointed out, the report entirely left out the clinical discovery of the effects of smoking on heart and lung disease, though this link was the "*most important therapeutic maneuver for most doctors treating lung and heart disorders*." [80] (Emphasis added.) Clinicians, in all likelihood, would not have left out that discovery, lending credence to the notion that Comroe and Dripps favored basic researchers when sending out their survey.

The Comroe Dripps Report is still cited as essential by those who wish to justify the use of sentient animals in basic research. (It has been the authors experience that these discussions usually occur outside the scientific literature hence another reason for the low number of citations for Comroe Dripps.) However, it was (in 1987, Smith questioned their conclusions [80]) and still is criticized by numerous scientists and clinicians for faults of methodology and bias. How reliable is the Comroe-Dripps analysis? Grant et al. observe that due to methodological flaws, the work by Comroe and Dripps

... would probably not meet today's standards for peer review. As Farrar observed, among the methodological problems " ... was a lack of clarity over whose opinions had been surveyed, how clinical advances were assessed and how a key article was defined." [81]

Grant et al. concluded that it takes about 17 years for basic research to have a clinical impact. More importantly:

Using the revised bibliometric protocol, we have shown in this study that ... between 2 per cent and 21 per cent of research was basic. This corroborates the findings of the clinical guidelines study that showed ... only 8 per cent of research was basic. These two findings are at odds with Comroe and Dripps finding that 40 per cent of all research articles judged to be essential for later clinical advance were not clinically oriented at the time of the study, thus undermining the evidence base that has, in the past, supported the increased funding of basic research. [[72]b]

Grant et al. concluded that Comroe Dripps was "not repeatable, reliable, or valid [82]." Strong words indeed. Additionally, Grant et al. did not address whether the basic science breakthroughs that were instrumental and were made using sentient animals could have been made without using them. If one is analyzing the importance of using sentient animals (Grant et al. were not), this is not an unimportant point.

More recently, others have also questioned the translation rate of basic research in general into clinically useful treatments. In 2003, Contopoulos-Ioannidis et al. quantified the translation rate of "highly promising" basic research into clinical applications. They published a study in the *American Journal of Medicine *that revealed of 101 basic research papers published in the high-profile journals *Nature, Cell, Science, the Journal of Biological Chemistry, the Journal of Clinical Investigation*, and *the Journal Experimental Medicine *between 1979 and 1983, twenty-seven led to randomized clinical trials and only five eventually gave rise to licensed clinical application [83,84]. They concluded that "[e]ven the most promising findings of basic research take a long time to translate into clinical experimentation, and adoption in clinical practice is rare [83]."

Contopoulos-Ioannidis et al. actually searched *all *the articles published in the above-mentioned journals between 1979 and 1983; a total of around 25,000. Crowley commented on this:

Of the 25,000 articles searched, about 500 (2%) contained some potential claim to future applicability in humans, about 100 (0.4%) resulted in a clinical trial, and, according to the authors, *only 1 (0.004%) led to the development of a clinically useful class of drugs (angiotensin-converting enzyme inhibitors) in the 30 years following their publication of the basic science finding*. They also found that the presence of industrial support increased the likelihood of translating a basic finding into a clinical trial by eightfold. Still, regardless of the study's limitations, and even if the authors were to underestimate the frequency of successful translation into clinical use by 10-fold, their findings strongly suggest that, as most observers suspected, *the transfer rate of basic research into clinical use is very low*[85]. Emphasis added.

The above casts severe doubt on the value of basic research in finding treatments and cures.

The Institute for Scientific Information (ISI) studied citation rates of papers published in journals indexed by the Institute between 1981 and 1985 and found that 55% of all articles were not cited within five years after publication [86]. The journals that ISI index are only the top ranked journals. The articles that appear in the lower ranked journals are not thought to receive as many citations as articles that appear in the top ranked ones. So the 55% figure is probably very high if all journals were considered. ISI also found that self-citation accounted for between 5% and 20% of all citations. The number of journals (science and nonscience) now numbers over 108,000 [86].

The actual results from years of rich funding to basic research is forcing some within the research community to acknowledge the failure of basic research to deliver on its promises [87]. Driven largely by this recognition, *translational medicine *has become a more frequent phrase in medical literature. Ioannidis, writing in the *Journal of Translational Medicine *presents a good example of this mindset: "There is considerable evidence that the translation rate of major basic science promises to clinical applications has been inefficient and disappointing [88]."

## How is progress measured?

Grant et al. expressed the expectations of funding medical research when they stated:

The United Kingdom spends over £1600 million a year on non-commercial biomedical and health services research. This research is funded either from the public purse, such as the NHS and the Medical Research Council, or medical research charities, such as the Wellcome Trust. *The tacit understanding is that the biomedical research these bodies support will lead to an eventual improvement in health*[89]. (Emphasis added.)

Many have questioned, however, if this funding is being properly directed and are asking for objective criteria for measuring the source of progress in medical practice [89-93].

Outgoing president Dwight Eisenhower seemed prescient when he warned in his last speech as president that the military-industrial complex was exerting too much influence in America's politics. The phrase *military-industrial complex *(meaning the marriage of the military with industry in general in order to obtain government money to fund projects the military desires) has been around ever since and currently most everyone understands what it means. What has been forgotten about Eisenhower's speech that day was that he had a similar warning about the influence the government had on scientific research [94]. A similar warning/analysis of government funded basic research is presented in *Nature *2008:

There is a growing disparity at the heart of biomedicine. In some ways, the field is experiencing a golden age: the quantity of basic research is shooting off the charts and budgets are far higher than they were two decades ago. Yet the impact of this research is growing at a much more modest rate: new cures and therapies are ever more expensive to develop and worryingly thin on the ground [95].

NIH has come under fire for funding basic research instead of more goal-oriented research [87,96-98]. Huge strides in basic research are not resulting in corresponding advances in the stated goal of NIH, which is "to reduce the burdens of illness and disability [99]." From 1998 to 2003, the budget of the National Institutes of Health doubled. The 2004 budget request was $27.9 billion. It is estimated that roughly 70% of NIH's research budget goes to basic science [97,98]. The percentage in the UK is about the same [100] and more recent numbers suggest the ratio has not changed [101-104].

But despite this infusion of cash, new chemical entities, the supposed fruits of basic research, went from having a 14% chance of success upon entering phase 1 trials to having an 8% chance of reaching the market [103]. Based on the conclusions of Contopoulos-Ioannidis et al [83] and Grant et al. [72] one might question the large percentage of research funds directed to a modality the results of which are responsible for such a small percentage of clinical breakthroughs. Chalmers:

Basic and applied research are both needed to find ways of protecting health, but the longstanding imbalance in the funding for these two broad spheres of biomedical research *cannot be defended in the light of what we know about their relative payback*[105]. (Emphasis added.)

In 2003, *JAMA *published a report prepared by a Clinical Research Roundtable (CCR) at the Institute of Medicine. The Institute of Medicine at the National Academies of Science convened a Clinical Research Roundtable in 2000 to analyze the success of basic research. They reported in 2003 that there is a "disconnection between the promise of basic science and the delivery of better health [102]." Rosenberg echoed the CRR when he called the notion that that the rapid growth in scientific publications and the increase in information about disease is resulting in better human health an "illusion." [104] The CRR also pointed out that clinical research receives about half the money that basic science receives [102], which is consistent with the 70% figure for basic research funding cited above. Similarly, a working group formed by the United Kingdom-based Academy of Medical Sciences expressed concern that clinical research, including large clinical trials, cohort studies, and meta-analyses was being ignored in favor of laboratory-based research [106].

Ioannidis has questioned the importance of basic research in resulting in better treatments [107]. Ioannidis addressed animal models and stroke. A study concluded that out of 1026 chemicals tested in animals, those chosen for clinical trials were not significantly different from the ones not chosen in terms of effect on infarct size [108]. In other words, the results from animal studies did not inform the choice for continuing to clinical trials. Ioannidis then states: "Evidence-based medicine does not seem to have penetrated basic and preclinical science, while basic and preclinical research is often performed in a clinical and methodological vacuum." [107]

There is clearly a great divide between the cold assessment of the current basic research paradigm's delivery on its promises and the rhetoric aimed at the public and lawmakers by the animal model community. For example, Sigma Xi: "An end to animal research would mean an end to our best hope for finding treatments that still elude us." [109]

## Basic research and the use of sentient animals

All of the above must be placed in the context of basic research using sentient animals. If basic research in the life sciences is questionable for finding cures, what about the questionable practice of using sentient animals in basic research? Rothwell:

In the current difficult financial environment for UK universities, only substantial increases in funding for practice-oriented research, preferably with full economic costing, will persuade them to take the research needs of the NHS more seriously. The intellectual and economic cases are strong, and the potential benefits are huge. *Indeed, most major therapeutic developments over the past few decades have been due to simple clinical innovation coupled with advances in physics and engineering rather than to laboratory-based medical research*. The clinical benefits of advances in surgery, for example, such as joint replacement, cataract removal, endoscopic treatment of gastrointestinal or urological disease, endovascular interventions (eg, coronary and peripheral angioplasty/stenting or coiling of cerebral aneurysms), minimally invasive surgery, and stereotactic neurosurgery, to name but a few, have been incalculable. Yet only a fraction of non-industry research funding has been targeted at such clinical innovation. How much more might otherwise have been achieved? [93] (Emphasis added.)

Rothwell goes on to say that much of the failure of basic research can be attributed to the use of animal models. He is not alone. Sydney Brenner who won a Nobel prize for research on *Caenorhabditis elegans *advocated for more research using *Homo sapiens *and called *Homo sapiens *"the model organism." [110]

Even the media has recognized the disconnect between basic research and treatments. Sharon Begley, writing in the *Wall Street Journal*:

"Patients," says immunologist Ralph Steinman of Rockefeller University, New York, "have been too patient with basic research."...Many of the brightest scientists have, therefore, plunged into the minutiae of roundworm genes and fruit-fly receptors, instead of human diseases. "Most of our best people work in lab animals, not people," says Dr. Steinman, who presents his case in a recent issue of the journal Cerebrum. "But this has not resulted in cures or even significantly helped most patients."... "Human experiments are much more time-consuming and more difficult than animal studies," says Rockefeller's James Krueger, whose human research includes trying to correlate gene activity and changes in immune-system cells with the progression of psoriasis. "There are also funding issues. It's much easier to write a successful grant proposal for animal experiments. Animals are homogeneous, and let you say 'aha!' in a neat, clean experiment." Humans, in contrast, are genetically and behaviorally diverse, making it hard to tell whether some aspect of their disease reflects the disease alone, their DNA, how they live -- or some messy permutation of all three [98].

It is difficult to say what percentage of basic biomedical research involves sentient animals as rats, birds, and mice need not be counted in accordance with the Animal Welfare Act. The best estimate we could find was from a 1985 publication from the Committee on Models for Biomedical Research, Board on Basic Biology (see figure 1 [111]). (Despite the NIH's public funding, more recent numbers are not readily available). [Table 1]

**Table 1:** Distribution of NIH Support of Extramural Research Among Humans, Laboratory Mammals, and Other Research Subjects, Expressed as Percentages of Total Dollars and of Total Projects and Subprojects.^a^

Subject	Fiscal Year Research Dollars, %	Extramural	Total Projects And Subprojects, %
Humans	1977	27.5	32.4
	1978	26.8	31.2
	1979	26.8	29.2
	1980	25.0	28.9
	1981	23.8	29.7
	1982	23.2	31.5
	1983	22.9	32.2
			
Mammals	1977	43.5	41.9
	1978	44.0	42.5
	1979	44.9	43.8
	1980	45.0	44.2
	1981	47.3	44.1
	1982	48.1	43.5
	1983	47.9	42.7
			
Other^b^	1977	29.4	25.6
	1978	29.3	26.3
	1979	28.2	27.0
	1980	29.8	26.9
	1981	28.9	26.0
	1982	28.7	25.0
	1983	29.2	25.1

^a^Unpublished information provided by Division of Research Resources, National Institutes of Health.

^b^This category includes invertebrates, nonmammalian vertebrates, bacteria, viruses, mathematical and computer simulations, and other subjects.

It appears that, on average greater than or equal to 50% of NIH extramural research dollars went to research involving sentient animals. According to the table, at least 45% went to research on mammals (most people consider mammals sentient and many even consider all vertebrates sentient [112-123]) and another 30% to research involving nonmammalian vertebrates and so forth (it appears that most people think at least some of these animals are sentient). Assuming some of the nonmammalian vertebrates are sentient, then the total is easily over 50%. Based on these numbers and NIH's predisposition to fund basic research, it appears feasible that at least 50% of extramural funding went to basic research on sentient animals.

In 1997, it was estimated that between 18 and 22 million animals were used in basic biomedical research in the U.S. and that about 85% of animals used were mice, rats, and birds [124]. In retrospect, that was probably a vast underestimate. Regardless, by 2000, this estimate had grown. A report prepared by the Library of Congress Federal Research Division estimated that the number of mice, rats, and birds used annually in the U.S. (in all areas) was more than 500 million [125]. The exact number of animals used in research is today, as was the case in 1997, unknown but the skyrocketing growth in the use of transgenic and otherwise genetically modified strains of mice alone suggests that the number must be very large. Madhusree Mukerjee, a former editor of *Scientific American*, stated that over 100 million transgenic mice were being used as of 2004 [124]. Five hundred million may in fact be on the low side. Has this apparently very significant increase in the use of animals yielded significant improvements or breakthroughs in the treatment of human illness? Apparently not. This has implications for using sentient animals in basic research

## Possible Objections

When the above is discussed with basic researchers who use sentient animals, several objections are forthcoming.

1. Discovering something new is very difficult and to describe it as "inefficient" implies there is a more efficient way of doing it. This is false. We must use animals.

This is fallacious for several reasons. First, just because something new is discovered does not mean the new discovery will have any meaning in terms of curing human disease. New discoveries are made everyday but, as the above studies report, that does not equate with new treatments. Second, there are numerous ways of conducting basic research and research designed to learn fundamental properties of living organisms such as humans. Using human tissue seems a very good methodology and has an excellent track record of leading to more knowledge about humans.

Third, perhaps the most damning analogy of inefficient basic research that uses sentient animals is *the glass bead game *popularized in Herman Hesse's book of the same name. Horrobin recently wrote an article about that very topic including the use of animals to search for knowledge about human drug and disease response.

A wonderful metaphor of much modern medical and pharmaceutical research can be found in the book entitled *The Glass Bead Game *by Herman Hesse. In this story, the leaders of the real world conspire with the brightest of scholars to create a magical state within a state, the isolated world of Castalia. Castalia recruits the most thoughtful and scholarly youths, educates them wonderfully well, and persuades them that the highest achievement of the human mind is to play the almost infinitely complicated and subtle 'glass bead game', an intellectual Olympics which challenges and stretches the most exceptional. The world of the game is beautifully refined and internally self-consistent. The only problems are that Castalia makes almost no contact with the real world, and that playing the game makes no contribution to real world issues [126].

Using sentient animals in basic research makes use of many resources. Funds that could go elsewhere, and researchers themselves, are commodities that are consumed.

Fourth, the question lumps together all kinds of basic research. Basic research in physics, for example, is hard and the best way to make new discoveries certainly appears to be doing basic research in the traditional way. But this does not imply that discovering new things about humans can or should be accomplished using sentient animals in basic research.

And finally, the claim that we cannot do it any other way is like Pascal's wager. "What do we have to lose by using animals?" The answer is, we lose what society would have received from research using the basic research modalities that do not include sentient animals; human tissue, *in silico *research, gene arrays, and so forth. Based on the above, it appears society has more to potentially gain from those nonanimal modalities than from using animals.

2. Your definition of basic research is wrong. Basic research *is *goal-oriented.

Based on the definition with which we began this paper, we respectfully disagree. But if basic research is synonymous with achieving goals then this points us back toward using animals as predictive surrogates for humans. We again direct the reader to a previous paper [1] and book [2] that address this issue in detail. If by *goal*, our critics mean increasing the amount of knowledge on the world, then this is a pointless tautology.

3. Society accepts using sentient animals as food, so to object to using animals in research is inconsistent.

We touched on this in when we discussed Spira's argument but will go into more detail here. The critic will get no argument from us that society is inconsistent. In fact society has been inconsistent in many ways in many different times. If society and the government waited for consistency nothing would ever change. However, the point we are making is that there are, at times, things that society, in sufficient numbers, objects to and is sufficiently disturbed by, that necessitate change regardless of the other inconsistencies implied by this. One obvious example is the abolition of slavery in the Deep South of the U.S. while simultaneously denying most blacks and women the right to vote. While eradication of the greater wrong should not be used to allow perpetuation of a lesser wrong, in fact, this is often the case. Yet it is equally important to note that the process of rectification is frequently iterative, and, as such, occurs as a series of changes over time.

The fact remains that society values certain resources, and some of those are not even sentient. The yew tree *Taxus brevifolia *is but one example. The anticancer medication Taxol was originally derived from the species of yew tree that was endangered and hence led to much discussion in society as to the value of the tree and its possible extinction versus using it to treat cancer. Robert Holton of Florida State University and Bristol Meyers solved the problem by discovering a way to use the common yew tree *Taxus baccata *to obtain a chemical that could then be modified to the active drug. Currently, the drug is produced by cell culture.

But prior to these breakthroughs, the issue was so contentious that the Native Yew Conservation Council (YewCon) was formed in the 1980's to address the problem. Some compared harvesting the endangered tree to slaughtering the buffalo [127-129]. (For the record, the authors disagree with the concern that values plants over cancer patients, but this simply illustrates the fact that different elements of society value things differently and that society *as a whole *may value something that individual members do not.)

4. Clinical research using humans is also flawed, as, frequently, the basic principles underlying the disease in question are not known.

Granted. But this assumes the basic principles can only be learned from using animals, as opposed to human tissues.

5. All research builds upon previous research that used animals; hence animals have been essential in all discoveries to date.

Fallacious. Just because A preceded B does not mean A caused B. There is a difference between animals being *necessary *for an advance as opposed to merely being *sufficient *for that advance. We touched on this when discussing further questions that need to be addressed. Moreover, even if animals used in basic research decades ago proved *necessary*, it does follow that the same is true today, with all the new technology and advances in science. If our critics want to take that line, then the burden of proof is on them to prove animals are currently necessary.

6. Regardless of how many breakthroughs of the past relied on animals, some did and that alone justifies the use of sentient animals in basic research.

There can be no doubt that some breakthroughs of the past were incumbent upon sentient animals. However, this objection does not consider current knowledge and technology available today that was not available in the past; the differences between questions asked in the past and those being asked today; the probability of finding treatments using sentient animals as opposed to other modalities; and the value society currently places on sentient animals as opposed to centuries or even decades ago.

7. When Thomas Edison was asked about all his failures in trying to invent a light bulb, he supposedly said that he had not failed 100 times (or 1000 times, sources vary on the exact number) but that he had succeeded in finding 100 ways that would not work and that when he had eliminated the ways that would not work, he will have found the one way that will work. That is what basic research is.

This is a cute little story and might even be true (again sources vary), but it has essentially nothing to do with our discussion. First, Edison was not spending resources that society valued beyond their monetary face value. Society values children more than orange juice and endangered plants more than those not endangered. Society does have a hierarchy of value. Scientists can spend resources that are largely of no or very little value to society, like common chemicals in a beaker, on the off chance something will result from it. But society mandates that researchers cannot spend, with impunity, resources it does value. Society values sentient animals more than inorganic materials.

Second, Edison was spending his own time and his own funds, and using resources society did not find to have inherent value. Therefore, society had essentially no legitimate grounds for telling Edison what to do with any of the above. Third, society did not fund Edison over other options. Fourth, Edison made no promises to society in exchange for its resources. His failures were largely irrelevant in that regard.

## Alternatives

There is nothing scientifically sacred about using sentient animals in basic research. Nonetheless, whenever we question the efficacy of such use we are met with the inevitable question: "How will we do basic research without using sentient animals?" Were this question not posed so seriously, we would suspect cynicism. But the questioner is serious so we will very briefly outline other methods available for basic research.

• The time-honored study of chemistry and physics has led to breakthroughs without which today we would still be practicing medicine circa the 19th century. Basic research in engineering and the physical sciences has historically led to advances in technology.

• *In vitro *research using human tissue.

• Bacteria, viruses, and fungi can be studied in order to discover basic cellular and genetic properties. Research using nonsentient, less complex organisms like *Drosophila *have given us the entire field of evo devo. As we mentioned, other organisms that could be studied include *E. coli, C. elegans*, *Brassica rapa, Saccharomyces cerevisiae, Phage Phi-X174, Dictyostelium discoideum*. This is a very partial list.

• Autopsies could be funded as non-goal oriented research as autopsies have historically led to many unsought discoveries and facts about the human body. New knowledge is still being generated by autopsies [130,131].

• The fields of mathematical and computer modeling offer ways to study complex systems but need funding.

• Basic research using human stem cells.

• Another important but oft-overlooked area of study is evolutionary biology. More emphasis needs to be placed on the study of evolution, the place of evolution in disease, and the implications of evolution for disease research and treatment.

The above is a very partial list. Eliminating the use of sentient animals in basic research would not lead to a dearth of basic research that needs funding. Ceasing to fund basic research using sentient animals would not help the NIH increase their application-funded to application-received ratio.

## Conclusion

Sir Ernst Chain, co-discoverer of penicillin, stated in 1970:

Science, as long as it limits itself to the descriptive study of Nature, has no moral or ethical quality, and this applies to the physical as well as the biological sciences. No quality of good or evil is attached to results of research aimed at determining natural constants, such as that of gravity or the velocity of light, or measuring the movements of stars, describing the kinetic properties of an enzyme, or describing the behaviour of animals (whatever our emotional attitude towards it may be) or studying the metabolic activity of a microbe, whether harmful or beneficial to mankind, or studying physiological function or pharmacological and toxic action. No quality of good or evil can be ascribed to studies aimed at the elucidation [of such questions] [132].

There can be no doubt that basic research has resulted in great breakthroughs in physics, chemistry, and biology. Almost by definition, in the early days of science, basic research was responsible for many, if not most, of the great discoveries. Today, we still see basic research generating a plethora of facts. The question we have posed is whether the controversial practice of using sentient animals in basic biomedical research is justifiable given society's distress about using such animals in such ways.

Sir Ernst's statement must be viewed in light of moral responsibilities that lie outside of science. Slavery, for example, is wrong even if slaves were to be used in scientific pursuits. Other issues also arise when one contemplates basic research. Philosopher Mary Midgley:

Sanctimonious obsessiveness needs to be publicly unmasked. It needs to be spelt out why an attempt understand desertification in Africa in order to resist it is not, just as such, at some deep level academically inferior to advance in theoretical physics. Something needs to be done here about the tendentious current use of words like 'basic' and 'fundamental' to describe any research which is not intended be useful. Trivial questions are still trivial, even when their answers are useless. Their uselessness cannot of itself transform them into fundamental ones [133].

Basic research is valuable for its own sake, even when treatments are not forthcoming. However, this value must be weighed against 1) other research that could be funded; 2) the cost, other than financial of performing the research; and 3) the value society places on sentient animals, even if society is at times inconsistent in applying that value.

In conclusion, we have shown that:

1. Society has expressed in open forums and via well-conducted surveys, its view that sentient animals should only be used in biomedical research that is likely to add treatments and cures or decrease the suffering of human patients. This position has been acknowledged by respected science journals.

2. Basic research has historically been justified based on its value in adding new knowledge to the world *not *on the basis of decreasing human suffering.

3. In the mid 20th century, this justification was threatened and researchers responded by connecting basic research to advances in medical science vis-à-vis the Comroe Dripps report.

4. Current research reexamining Comroe Dripps and the contribution of basic research in general to discovering new treatments and cures has revealed that there currently exists a low probability that basic research in general will lead to cures for human disease. This does not contradict the fact that historically basic science research in general and basic science research using sentient animals specifically resulted in breakthroughs in biomedical research. The times, available methods, and questions have changed.

5. According to figures from the NIH, basic biomedical research receives more funding than all other forms of research, uses animals more often than not, and many if not most of these animals would be classified by society in general as sentient. There is a high probability that such use will, by the very fact that the animals will be confined in an unnatural environment, cause pain and suffering.

Based upon our interpretation of data gained from research published in peer reviewed journals, public opinion polls, and comments reflecting the aforementioned in the scientific literature, we conclude that society does not condone using sentient animals in basic research.

## Competing interests

The authors declare that they have no competing interests.

## Authors' contributions

Both authors contributed equally and have read and approved this manuscript.

## About the authors

Ray Greek, MD completed medical school at the University of Alabama in Birmingham and a residency in anesthesiology at the University of Wisconsin-Madison. He taught at the University of Wisconsin and Thomas Jefferson University in Philadelphia. He is now the president of the not-for-profit Americans For Medical Advancement http://www.AFMA-curedisease.org.

Jean Greek, DVM completed veterinary school at the University of Wisconsin-Madison and a residency in dermatology at the University of Pennsylvania. She taught at the University of Missouri and is now in private practice.
